# Genome Sequences of Strain ATCC 29281 and Pin and Northern Red Oak Isolates of *Lonsdalea quercina* subsp. *quercina*

**DOI:** 10.1128/genomeA.00584-14

**Published:** 2014-06-12

**Authors:** Jorge Ibarra Caballero, Marcelo M. Zerillo, Jacob Snelling, Whitney Cranshaw, Christina Boucher, Ned Tisserat

**Affiliations:** a Department of Bioagricultural Sciences and Pest Management, Colorado State University, Fort Collins, Colorado, USA; b Department of Computer Science, Colorado State University, Fort Collins, Colorado, USA

## Abstract

Two bacteria identified as *Lonsdalea quercina* subsp. *quercina* were isolated from oak trees showing symptoms of drippy blight. Here, we present their draft genome assemblies, as well as that of the type strain of this species. To our knowledge, these are the first published genome sequences of this subspecies of *Lonsdalea quercina.*

## GENOME ANNOUNCEMENT

*Lonsdalea quercina* (Hildebrand and Schroth 1967) Brady et al. 2012 (1), first described as *Erwinia quercina* (2) and later as *Brenneria quercina* (3), is associated with bacterial gummosis and twig necrosis of several oak (*Quercus*) species (drippy blight disease) (2, 4). We recovered two isolates, NCCB100489 and NCCB100490, in Colorado from a symptomatic pin oak (*Quercus palustris* Muench) and a northern red oak (*Quercus rubra* L.), respectively, which exhibited small cankers and gummosis at the Kermes scale (*Allokermes galliformis* Riley) feeding sites (5). We sequenced the genomes of these isolates and the genome of the *L. quercina* subsp. *quercina* type strain, ATCC 29281, which was originally collected from a diseased California live oak (*Quercus agrifolia* Nee) in California (2). Sequencing with 100 cycles of paired-end reads using the Illumina HiSeq sequencer at the University of Southern California (USC) Epigenome Center yielded more than 22.7 (NCCB100489), 23.8 (NCCB100490), and 32.9 (ATCC 29281) million 100-base-long reads for each paired end of each one of the three isolates. The assemblies were performed using Mira 4 Orc4 (6).

We obtained total assembly lengths of 3,848,371 bp, 3,847,844 bp, and 3,850,073 bp, contig numbers (and largest contig sizes in base pairs) of 35 (707,372), 35 (512,358), and 37 (557,855), *N*_50_ sizes of 334,484 bp, 279,081 bp, and 282,816 bp, and G+C contents of 55.6, 55.6, and 55.1% for strains NCCB100489, NCCB100490, and ATCC 29281, respectively. We annotated the assembled genomes using the RAST server (7) and detected 3,398 (NCCB100489), 3,400 (NCCB100490), and 3,460 (ATCC 29281) coding sequences representing 475, 475, and 472 subsystems, respectively. To further check the robustness of the assemblies, we performed a BLASTn (8) search for genes from *L. quercina* isolates that had previously been deposited in the GenBank database. In the ATCC 29281 genome assembly, the 16S rRNA gene was found to be 99% similar to the same gene reported in *L. quercina* subsp. *quercina* strain LMG 2725, while the *atpD*, *gapA*, *gapDH*, *infB*, *recA*, and *rpoB* genes were 100% identical and *dnaJ*, *gyrB*, and *ompA* were 99%, 99%, and 97% similar, respectively, to the reported sequences in ATCC 29281 deposited into the GenBank by other groups. All these mentioned genes were identical to each other in the NCCB100489 and NCCB100490 genome assemblies, but these isolates had lower similarities to the corresponding sequences of 16S rRNA (99%), *atpD* (95%), *gapA* (97%), *gapDH* (98%), *infB* (96%), *recA* (97%), *rpoB* (98%), *dnaJ* (95%), *gyrB* (98%), and *ompA* (93%) in ATCC 29281. Nevertheless, a multilocus (16S, *gyrB*, *atpD*, and *infB*) phylogenetic tree based on the Bayesian inference (9) grouped NCCB100489, NCCB100490, and ATCC 29281 in an *L. quercina* subsp. *quercina* cluster, distinct from *L. quercina* subsp. *iberica* and *L. quercina* subsp. *britannica* isolates (data not shown). Biological testing is needed to explore the pathogenicity and host range differences of these and other *L. quercina* strains.

### Nucleotide sequence accession numbers.

These whole-genome shotgun projects have been deposited at DDBJ/EMBL/GenBank under accession no. JIBO00000000, JIBP00000000, and JIBQ00000000. The versions described in this paper are the first versions, JIBO01000000, JIBP01000000, and JIBQ01000000.
